# *Odyssey*: a semi-automated pipeline for phasing, imputation, and analysis of genome-wide genetic data

**DOI:** 10.1186/s12859-019-2964-5

**Published:** 2019-06-28

**Authors:** Ryan J. Eller, Sarath C. Janga, Susan Walsh

**Affiliations:** 1 Department of Biology, Indiana University-Purdue University Indianapolis, 723 W. Michigan Street, Indianapolis, IN USA; 2 Center for Computational Biology and Bioinformatics, Indiana University School of Medicine, 5021 Health Information and Translational Sciences (HITS), 410 West 10th Street, Indianapolis, IN USA; 3 Department of Biohealth Informatics, School of Informatics and Computing, Indiana University-Purdue University, Indianapolis, IN USA

**Keywords:** Imputation, Phasing, Pipeline, Genome-wide-association study, Admixture, Odyssey

## Abstract

**Background:**

Genome imputation, admixture resolution and genome-wide association analyses are timely and computationally intensive processes with many composite and requisite steps. Analysis time increases further when building and installing the run programs required for these analyses. For scientists that may not be as versed in programing language, but want to perform these operations hands on, there is a lengthy learning curve to utilize the vast number of programs available for these analyses.

**Results:**

In an effort to streamline the entire process with easy-to-use steps for scientists working with big data, the *Odyssey* pipeline was developed. *Odyssey* is a simplified, efficient, semi-automated genome-wide imputation and analysis pipeline, which prepares raw genetic data, performs pre-imputation quality control, phasing, imputation, post-imputation quality control, population stratification analysis, and genome-wide association with statistical data analysis, including result visualization. *Odyssey* is a pipeline that integrates programs such as PLINK, SHAPEIT, Eagle, IMPUTE, Minimac, and several R packages, to create a seamless, easy-to-use, and modular workflow controlled via a single user-friendly configuration file. *Odyssey* was built with compatibility in mind, and thus utilizes the Singularity container solution, which can be run on Linux, MacOS, and Windows platforms. It is also easily scalable from a simple desktop to a High-Performance System (HPS).

**Conclusion:**

*Odyssey* facilitates efficient and fast genome-wide association analysis automation and can go from raw genetic data to genome: phenome association visualization and analyses results in 3–8 h on average, depending on the input data, choice of programs within the pipeline and available computer resources. *Odyssey* was built to be flexible, portable, compatible, scalable, and easy to setup. Biologists less familiar with programing can now work hands on with their own big data using this easy-to-use pipeline.

**Electronic supplementary material:**

The online version of this article (10.1186/s12859-019-2964-5) contains supplementary material, which is available to authorized users.

## Background

Genome-wide association studies (GWAS) have grown in popularity thanks to the increased availability of genome-wide data and sequence information. GWAS, while successful at identifying candidate variants, has also been aided by imputation methods [1–3] that increase coverage and allow for increased sensitivity. Imputation is often performed to fill in the genomic gaps, increase statistical power, and to “standardize” datasets so they can be combined with others that are genotyped with different arrays [4]. Over the last few decades several reference datasets have become available to use for imputation, such as the international HapMap Project in 2003 [5], the 1000 Genome Project in 2015 [6], the Haplotype Reference Consortium (HRC) in 2016 [7], and the more recently announced All of US Research Program currently being conducted by the NIH in 2018 [8]. Increasing the number and diversity of reference panels allow for increased flexibility in how imputation is performed for a particular sample set. Van Rheenen et al., 2016 has shown that custom reference panels that combine an existing reference panel with sequence data collected from a subset of individuals within their analysis cohort may also increase imputation accuracy [9].

Current imputation options include the popular free-to-use imputation servers such as the Michigan Imputation Server (https://imputationserver.sph.umich.edu/) and the Sanger Imputation Server (https://imputation.sanger.ac.uk/), which provide an online solution to imputation. There are also offline solutions such as the Michigan Imputation Server Docker [10], and imputation packages such as Python’s Genipe [11]. The strengths of online solutions are that they normally require no setup and are easy to use. However, a major drawback is that they require data to be sent off-site (albeit via a secure SFTP or SCP connection in the cases of the Sanger and Michigan Imputation Servers), which may or may not be possible for a researcher due to ethical or legal constraints. As with most online servers users may need to sit in a queue before their job is run, and users are often restricted by analysis options, such as the choice of phasing/imputation programs as well as the reference panels the sites support. At the time of writing, both the Sanger and Michigan Imputation Servers support three main panels: the HRC, 1000 Genomes (Phase 3), and the CAAPA African American Panel [12]. Sanger also provides access to the UK10K dataset, which is currently unsupported by the Michigan Imputation Server. It is important to note that apart from Sanger’s option of imputing with a combined UK10K dataset and 1000 Genomes Reference panel, the online solutions do not give much flexibility if the user wishes to combine several reference panels or integrate collected data into a custom reference set to enrich the imputation. Users must then opt for offline solutions, such as the Michigan Imputation Server Docker image and Python Packages such as Genipe, that do not require data to be sent offsite and provide considerably more flexibility in the imputation analysis as they allow custom reference datasets to be installed. However, an issue of using offline solutions is that they need to be configured by the user, which may not be straightforward due to the many programs these pipelines require as well as their interconnected library dependencies.

While imputation is the main goal of all these platforms, it is imperative that data must be formatted properly before submitting it through phasing and imputation. Furthermore, quality control measures should be enacted to achieve the highest possible imputation accuracy. While a researcher should know what quality control measures they would like to use for imputation, there are inconsistencies between different programs and their default settings. The established online imputation servers perform some filters for minor allele frequency, duplicated variant detection, and VCF integrity, but most of the data cleanup is left to the user. While this data prep must be done offline, most of these platforms provide a thorough walk-through on how to implement these steps. It is also worth noting that the offline docker solution, which is similar to the Michigan Imputation Server, provides guidance with quality control but like its online counterpart does not perform it automatically. Thus, the responsibility of proper data preparation falls largely on the user and their ability to find acceptable imputation quality control thresholds, such as those found in Manolio et al., 2007 [13]. In addition to cleaning the data, the user is expected to provide data in a compatible format for the imputation workflow, which is normally a VCF (for the Sanger and Michigan Imputation Servers and docker image) or a PLINK .bed/.bim/.fam (for the Genipe package). While most commercial genome array software, such as Illumina’s Bead Studio or Affymetrix’s Power Tools, perform these conversions, the user must still rectify any genome array compatibility issues, such as remapping incompatible sample data to the same genome build used by the imputation reference panel.

Genome-wide association studies that use probabilistic imputation data or dosage data require a considerable number of programs for the analysis to be run. Currently, only PLINK 2.0 [14] and SNPTEST [3, 4, 15] are capable of performing such analyses, short of writing a custom script. It is important to note that additional programs accept dosage data, but subsequently hard-call (i.e. probabilistically round) genotypes that are used in downstream analysis. While analyzing hard-called data is a valid strategy, data is ultimately being altered and may alter study outcomes. In addition to having few analysis programs that can analyze dosage data, it is often cumbersome and time consuming to input data into these programs. Dosage data often needs to be concatenated or merged (as imputation is normally done in segments) and then converted into a format accepted by PLINK 2.0 or SNPTEST in a manner that does not alter the data as previously described. Further complicating the matter of compatibility is the continual evolution of dosage data formats, such as Oxford’s .bgen and PLINK’s .pgen, since programs may not accept both file formats or even certain version iterations of a particular filetype. Due to the aforementioned issues, the transition between imputation and data analysis is the largest hurdle to analyzing imputation data and is probably an area in the largest need of improvement in imputation analysis workflows.

Admixture considerations while performing a GWAS lie primarily in performing a separate stratified analysis using ancestry informative programs such as the model-based Admixture [16] or PCA-based Eigensoft [17] programs, and therefore require knowledge of these additional programs to account for population stratification prior to GWAS analyses. Of course, another option is to perform the analysis via a program that supports a linear mixed model (LMM) and therefore does not require pre-ancestry testing, such as BOLT-LMM [18] or GEMMA [19], which takes ancestral interactions into account during the association analyses [20]. However, this may not be the desired algorithm of choice for most GWAS.

While much effort is expended on performing the analyses, it is essential to remember that dissemination of the results in an easy to understand manner is equally as important. Result condensation and visualization via charts, graphs, and summary tables is therefore important in any imputation analysis workflow. Advanced R plotting packages, such as Plotly [21], allow close integration with association analyses, providing users with interactive Manhattan and Quantile-Quantile (QQ) plots that give an overview of the GWAS results. Plotly data visualizations are also invaluable when assessing admixture-based PCA plots since the plots are often three-dimensional and more easily to interpret as dynamic images. At present, incorporation of data visualization into a GWAS pipeline is not present on any previously published workflows.

Genipe is one of the first to successfully integrate many of the imputation and GWAS workflow steps, as described above, into a single, easy-to-use package. The Python package is designed to facilitate the transfer of data through phasing, imputation, and various analyses using a variety of program dependencies such as PLINK, SHAPEIT, IMPUTE, and Python analysis packages as well as various custom analysis scripts. Similar to other imputation platforms, Genipe lacks built-in pre-imputation quality control measures, instead outsourcing quality control to the user via recommendations in the user manual. In addition, the program gives the option of running logistic and linear analyses, but fails to assess sample admixture, which would require the user to refer to external admixture analysis programs prior to running these analyses. However, Genipe does give the option of running an LMM, which historically has shown more success than naive logistic and linear analyses for admixed samples [22]. In addition, the program does not provide ways to visualize the association results, which would have provided a nice complement to its large repertoire of analysis options. Finally, while it is easy to setup Genipe’s Python-based framework, it does require the user to manually install and configure several of its dependencies.

Essentially, it would be beneficial from a time and resource perspective to have an imputation solution that can leverage the easy setup of online imputation servers with the flexibility of local imputation packages. Being able to control the workflow’s options and automations steps from a single configuration file would also be an advantage over programs that require the user to refer to a lengthy user-manual describing the necessary flags needed to implement a program feature. Here we describe a flexible and easy-to-use local pipeline that not only phases and imputes data, but also automates data preparation, organization, quality control, admixture and association analysis, and visualization of genome-wide data. This pipeline was designed to be compatible with all major operating systems and is also scalable, having the ability to leverage the computational power of HPS, facilitating parallelization and reducing GWAS run time from start to finish.

## Methods and implementation

Several obstacles of many pipelines that contain multiple dependencies is portability, compatibility, and in the case of this resource intensive process, scalability. *Odyssey* attempts to address each of these issues by utilizing Singularity [23], which is similar to the commonly used Docker container solution (https://www.docker.com/). All of *Odyssey’s* dependencies save two (IMPUTE4 due to licensing restrictions and GNU-Parallel due to technical limitations), are packaged into a Singularity container, which is contained within the *Odyssey* Github repository. Therefore, running *Odyssey* is as easy as installing Singularity on the host system allowing for increased portability. Since Singularity can be run on all major operating systems including Linux, MacOS, and Windows, this allows *Odyssey* to be compatible on the same systems. Unlike Docker, Singularity was created with High-Performance Systems in mind, and thanks to its unique handing of user security settings, is employed on many HPS around the world allowing *Odyssey* to scale from small desktops to large cluster computing systems.

*Odyssey* is primarily a collection of Bash and R scripts housed within a Github repository that are controlled by a single configuration text file. Researchers who wish to use the main functions of *Odyssey* would thus only need to interact with a single file that contains all the “flags” that affect how *Odyssey* behaves. Whereas other programs are controlled via command line by specifying flags and their subsequent options, *Odyssey*, explicitly states its options (as well as a small description of its purpose), which partially eliminates the need to refer to a user manual.

**Fig. 1:**
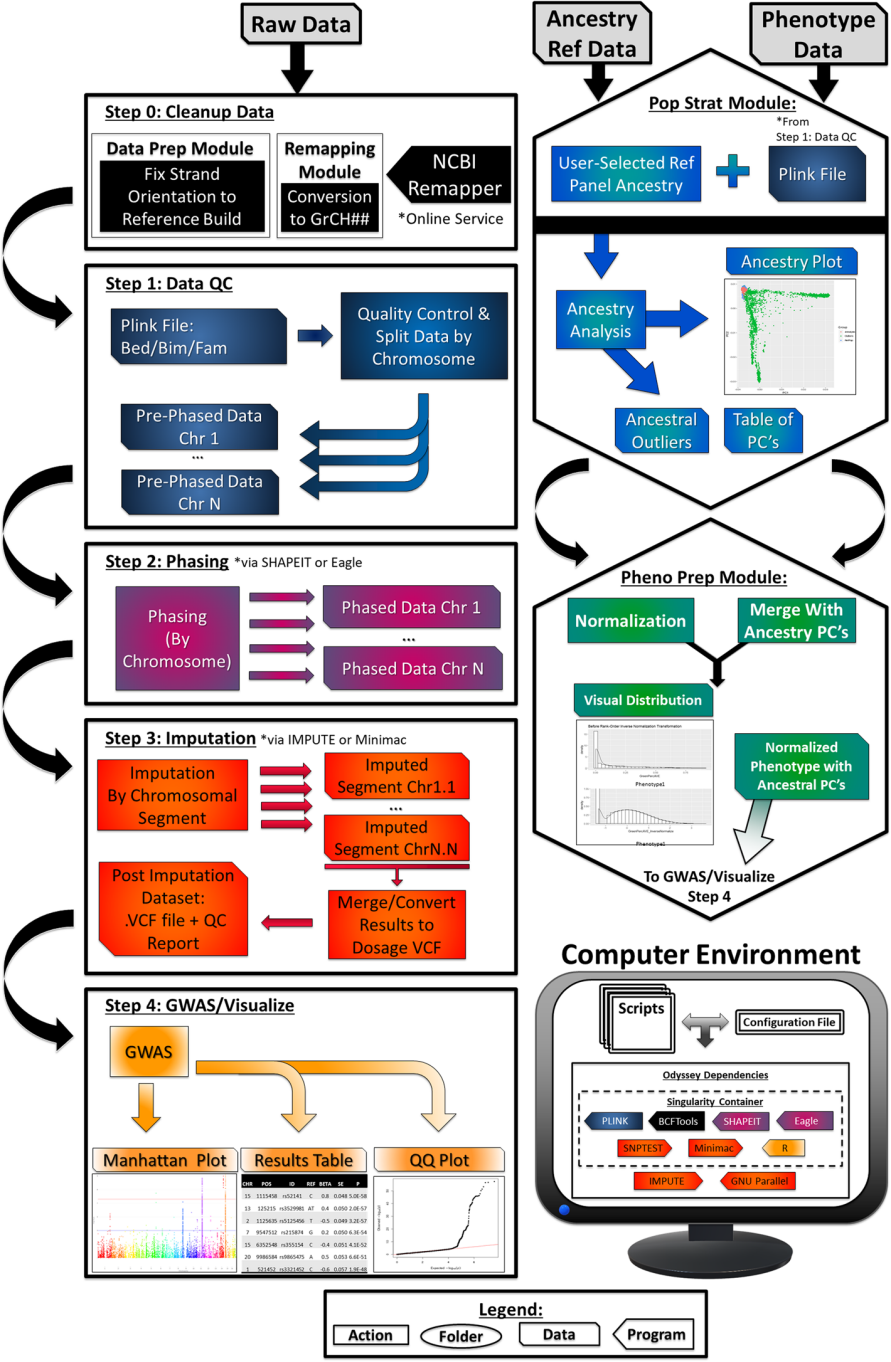
*Odyssey* Workflow. *Odyssey* performs 4 steps after data cleanup: Pre-Imputation Quality Control, Phasing, Imputation, and GWAS Analysis. Data can be easily removed from the pipeline at the ends of each major step. A Population Stratification and Phenotype Prep Module are provided, which assists in the removal of ancestral backgrounds deemed unwanted though a PCA-based approach and normalizing phenotypes

*Odyssey* also relies on a set of dependent programs, which are all installed and configured (save IMPUTE4 and GNU-Parallel) on startup, to perform the pipeline’s main tasks. These bioinformatic programs include PLINK [14] and BCFTools [24] to perform quality control and analysis, SHAPEIT2 [25] and Eagle2 [26] for phasing, IMPUTE4 [3, 27] and Minimac4 [10] for imputation, SNPTEST [3, 4, 15] for post-imputation quality control reporting, R as a platform for visualization and population stratification analysis, and GNU-Parallel [28] for increasing throughput. The pipeline is divided into the following main steps (see Fig. 1).

Step 0 provides a range of data cleanup options designed to take genotype data from a sequencer and prepare it for imputation and downstream analysis. The input criteria for *Odyssey* is a PLINK .bed/.bim/.fam. While there are a range of genotyping platforms, Illumina and Affymetrix were used as a starting point for which there are tools (i.e. BeadStudio with the PLINK Plugin, and Affymetrix Power Tools respectively) to convert raw array data into PLINK format. The Remapping Module in Step 0 gives the option of remapping input data to the genome build used in the imputation reference panel by utilizing NCBI’s Coordinate Remapping Service (https://www.ncbi.nlm.nih.gov/genome/tools/remap). The Data Prep Module provides the option of using BCFTool’s “fixref” plugin to correct strand orientation errors on the input data so that it matches a given reference dataset, which helps improve imputation as well as reducing the chance of getting an imputation error downstream. Both modules within Step 0 are optional and may be used if needed.

Step 1 calculates quality control metrics (including missingness, minor allele frequency, relatedness, etc.) with PLINK, and visualizes the data to better inform the use of the nature of the dataset. In addition, *Odyssey* provides the option to filter out variants that do not pass the default thresholds, which while set based on current practices [13], can be modified from *Odyssey’s* configuration file. Quality controlled data is separated by chromosome and sent to Step 2 where it is phased with either SHAPEIT or Eagle, depending on user preference. Like most other imputation pipelines, *Odyssey* supports the phasing, imputation, and analysis of the X chromosome. At the end of Step 2, an internal check is performed to determine whether all chromosomes were phased properly. If a chromosome failed imputation, *Odyssey* displays which chromosome failed, why it failed (by returning the phasing error message), and can be set to re-phase the offending chromosome/s.

In Step 3 phased chromosomal data is imputed with IMPUTE or Minimac, depending on user preference, in chromosomal segments to ease the computational burden of imputation. Following imputation, another error check, similar to the error check following Step 2, is performed to check for imputation errors and provides guidance on fixing offending segments. Once all the chromosomal segments are imputed, a post-imputation quality control check is run, where poorly imputed variants are filtered out based on a user-specified IMPUTE INFO or Minimac R^2^ metric. The resulting files are converted and merged into a dosage VCF-4.3 with PLINK and BCFTools, which can be loaded into most major analysis programs including PLINK and SNPTEST. *Odyssey* also provides a “Custom Reference Panel Creator Module”, which semi-autonomously takes several user-provided reference panels (in .hap and .legend formats, which can be created from running VCF or PLINK files through SHAPEIT) and merges them together via IMPUTE to create a custom imputation reference panel. In this way users are not limited to using the default 1000 Genome Phase 3 Reference Panel that is downloaded and can thus use *Odyssey* to tailor their imputation runs to their own data.

Step 4 uses the dosage data calculated in Step 3 in addition to a user-provided phenotype file to perform a GWAS using PLINK, whose results are parsed, analyzed, and visualized in R via a summarized table, a Quantile-Quantile plot, and an interactive Manhattan plot using several R packages. In addition, a Population Stratification Module can be run prior to performing the GWAS, which visualizes the ancestral background of cohort individuals. Then, users can either incorporate this ancestry information into the final GWAS as a covariate or exclude individuals who lie outside of an acceptable ancestral background. This exclusion method is accomplished via an Eigensoft-like method [17] in which a reference set (e.g. the 1000 Genomes reference data) is combined with cohort data in a Principal Component Analysis to establish an X-dimensional centroid that identifies the ancestry the user wishes to retain. Outliers that fall outside of the X-dimensional centroid are determined based on a specified standard deviation or inter quartile range cutoff. Unlike Eigensoft, the exclusion method performed by *Odyssey* only occurs once as opposed to Eigensoft’s iterative exclusion method.

Since imputation creates many files, *Odyssey* organizes all the data by grouping it into 6 folders (one folder for each step including a summary project folder that contains project meta data collected from each step) and provides a single dosage VCF.gz output that can be manipulated and viewed with programs such as PLINK, SNPTEST, or BCFTools. *Odyssey* also provides support for archiving multiple imputation runs and GWAS analyses since data is organized in the 6 folders within discrete “Project” directories. In this way a user may run multiple GWAS analyses or Imputation runs without worrying about data being overwritten. As an added benefit these modularized projects allow the user to zip and extract data at the end of each step. In this way, raw project data or the summarized results folder can be easily shared with collaborators and even integrated within their *Odyssey* pipeline for further analysis.

## Results and discussion

*Odyssey* provides a User Manual, a tutorial, and a publicly available HGDP dataset [29] (http://www.hagsc.org/hgdp/files.html) to illustrate a sample workflow for new users. Benchmarking was conducted on Indiana University’s large memory HPS, Carbonate. Carbonate contains 72 Lenovo NeXtScale nx360 M5 server compute nodes containing 12-core Intel Xeon E5–2680 v3 CPUs and 256 GB of RAM, in addition to 8 large-memory compute nodes containing 512 GB of RAM. RAM and CPU usage metrics were collected using the collectl utility (http://collectl.sourceforge.net/). To provide a baseline estimate of the resources needed by *Odyssey* for an imputation job, benchmarking was conducted using 3 CPU’s when applicable.

The Human Genome Diversity Project (HGDP) dataset of 940 individuals with 542 K genetic markers (after quality control) was used in a SHAPEIT-IMPUTE and Eagle-Minimac workflow to show *Odyssey’s* performance metrics. A breakdown of these benchmarks for each step can be found in Additional file 1 Table S1 and S2, in addition to real-time analyses in Additional file 1: Figure S1-S11. To summarize, all 940 individuals were cleaned, pre-imputation quality-controlled, phased, imputed, post-imputation quality controlled, analyzed (by performing a linear regression on the dosage data and randomly generated phenotypic data), and visualized within 8 h when using SHAPEIT-IMPUTE and within 3 h when using the Eagle-Minimac workflow. Performing the optional Population Stratification add-in using the HGDP dataset and the 1000 Genomes reference set to remove non-European individuals took approximately 20 min. One of the major steps, imputation, using the 1000 Genomes Phase 3 reference panel provided by IMPUTE (https://mathgen.stats.ox.ac.uk/impute/1000GP_Phase3.html), imputed approximately 40 M (post-QC) genotypes from 542 k input genotypes in approximately 20 min by running the SHAPEIT-IMPUTE workflow on Carbonate’s hyperthreaded Xeon E5–2680 CPU’s, which performed 100 to 200 concurrent jobs. Conversely, when running the Eagle-Minimac workflow on the same hardware, using the same input genotypes, and a 1000 Genomes Phase 3 reference panel provided by the Minimac4 website (https://genome.sph.umich.edu/wiki/Minimac4), imputation took 45 min and 25.4 M (post-QC) variants were imputed. Therefore, in this comparison, although the choice of the Eagle-Minimac workflow was faster, the total number of variants available post QC for GWAS was only 64% of the total variants available when implementing the SHAPEIT-IMPUTE workflow under a set 0.3 INFO score threshold. This disparity could be due to the fact that imputation quality control cutoffs need to be adjusted when using alternative imputation programs and that reference panels are curated differently (e.g. some variants may be taken out of a reference panel to simplify the imputation analysis). These factors are all important considerations when choosing a workflow to help maximize the effectiveness of an imputation analysis. However, when all these aspects are held equal as shown by Liu et al., 2015 [30], the accuracy differences between imputation workflows, specifically IMPUTE v Minimac, are small.

While a direct analysis with the popular online solutions, such as the Sanger Imputation Server, could not be easily measured (due to the randomness of queue wait times), in general a small dataset (N~ 900 with 550 K markers) could be submitted to Sanger and returned within similar time frames. This is expected due to the underlying programs that runs *Odyssey*, the Sanger and Michigan Imputation Server, and Genipe are similar, if not identical, and thus have similar time and resource requirements. Thus, in general the speed of the analysis will primarily depend on the hardware available to the user. While the runtime of the analyses will be similar, the setup time of these pipelines vary depending on the amount of data prep, the configuration of the imputation solutions, and the input of imputation options. *Odyssey* attempts to minimize setup time by employing modules that streamlines the data prep process, utilizing Singularity, which minimizes the time needed to configure the pipeline, and using a configuration file, which centralizes control of the pipeline and minimizes the need to constantly refer to a reference manual to lookup program options.

In the future, *Odyssey’s* capabilities will be further improved via implementation into domain-specific language (DSL) implicit frameworks such as Snakemake [31] and by continuing to explore routes to optimize the pipeline to save time and space.

## Conclusion

In conclusion, *Odyssey* allows quick and easier access to genome imputation by scientists who seek a local pipeline that is easy to setup, offers the flexibility to accommodate highly customizable analyses, and accommodates those who may not be allowed to outsource data to imputation servers. *Odyssey* attempts to take the best parts of the previous local and cloud imputation solutions and combine them into a portable, compatible, and scalable pipeline that offers a default simple analysis option for those wanting a simple analysis, or a highly customizable advanced analysis options for those looking for more complex analysis. Using modular and portable project directories, *Odyssey* is built to maximize collaborations as project data and results may be ported from one research group to another. Ultimately, *Odyssey* condenses a difficult workflow into a fast and easy-to-use pipeline that will benefit and complement biologists working with big data from multiple admixed cohorts.

### Availability and implementation

The *Odyssey* pipeline is an open source suite scripts that can be easily setup on any Linux environment and executed with their Linux program dependencies of PLINK, SHAPEIT, Eagle, IMPUTE, Minimac, BCFTools, SNPTEST, and R, which are handled automatically via the Singularity container solution, which requires a Singularity installation. However, an installation, which does not use Singularity, is also possible. The program was implemented primarily with Bash and R and was tested under Linux on a desktop and an HPC.

## Additional file

Additional file 1: Two tables that list the summarized benchmarking results and eleven figures giving an in-depth look at each benchmarking step. The table show a summarized benchmark of all eight of *Odyssey’s* steps using the HGDP dataset on a SHAPEIT-IMPUTE (**Table S1**) and an Eagle-Minimac (**Table S2**) workflow. Eleven figures follow the table which provide a visual assessment of the CPU utilization (figure on the left) and RAM usage (figure on the right) for each of the eight pipeline steps for each of the workflows summarized in the tables.(PDF 811 KB). (DOCX 971 kb)

## Data Availability

While reference data is downloaded automatically by *Odyssey*, the following links will download the 1000 Genome reference panel used for phasing/imputation: (https://mathgen.stats.ox.ac.uk/impute/1000GP_Phase3.html) (ftp://share.sph.umich.edu/minimac3/G1K_P3_M3VCF_FILES_WITH_ESTIMATES.tar.gz). The HGDP dataset, which is used for benchmarking, is also provided with *Odyssey*. However, it may also be downloaded from here: http://hagsc.org/hgdp/files.html. *Odyssey* is freely available for non-commercial use (under GNU General Public License v3) at https://github.com/Orion1618/Odyssey.git

## Abbreviations

GWAS: Genome wide association study
HGDP: Human Genome Diversity Project
HPS: High Performance System
HRC: Haplotype Reference Consortium
