# Supplementary material for: Odyssey: a semi-automated pipeline for phasing, imputation, and analysis of genome-wide genetic data
Source: BMC Bioinformatics. 2019 Jun 28;20:364. doi: 10.1186/s12859-019-2964-5 (PMC6599316; doi:10.1186/s12859-019-2964-5)
Supplement: Supplementary file 1 — Two tables that list the summarized benchmarking results and eleven figures giving an in-depth look at each benchmarking step. The table show a summarized benchmark of all eight of Odyssey’s steps using the HGDP dataset on a SHAPEIT-IMPUTE (Table S1) and an Eagle-Minimac (Table S2) workflow. Eleven figures follow the table which provide a visual assessment of the CPU utilization (figure on the left) and RAM usage (figure on the right) for each of the eight pipeline steps for each of the workflows summarized in the tables.(PDF 811 KB). (DOCX 971 kb) [file 12859_2019_2964_MOESM1_ESM.docx]

***Odyssey*: A semi-automated pipeline for phasing, imputation, and analysis of genome-wide data**

**Supplementary Material**

Eller, R.J. Janga, S.C. Walsh, S.

**Table S1 and S2.**Benchmarks for *Odyssey*. Benchmarks were conducted on an admixed HGDP sample set of 940 individuals (542K variants after quality control) with a 1000 Genome Phase 3 Reference dataset (80M variants) on a High-Performance Cluster using a SHAPEIT-IMPUTE and a Eagle-Minimac workflow. While Odyssey can be parallelized almost infinitely, benchmarks were conducted on 3 CPU throughout the pipeline unless programs were unable to utilize the additional cores or the use of additional cores would be unlikely to increase performance. Benchmarks performed for each step are listed and illustrate the maximum amount of RAM and time required to complete each step. Since phasing and imputation can be massively parallelized their benchmarking times are divided into “Time per Job,” the time it took to complete a single job for a benchmarking sample for the given step and “Time per Step,” the total amount of time it took to complete the entire step for the entire HGDP dataset running on up to 150 concurrent jobs on the HPS. Two tables are shown for 2 specific workflows, the SHAPEIT-IMPUTE (left) workflow and also Eagle-Minimac (right).

| Step | Max Memory Required | CPU's Used | Benchmarking Sample | Time per Job on HPS | Time per Step on HPS |
| --- | --- | --- | --- | --- | --- |
| Step 0 - Clean-up | 0.9 GB | 1 | HGDP Dataset | 0:18:15 | 0:18:15 |
| Step 1 - Pre-QC | 0.1 GB | 1 | HGDP Dataset | 0:01:07 | 0:01:07 |
| Step 2 – Phase* | 1.9 GB | 3 | Chr 1 | 0:24:22 | 0:24:22 |
| Step 3a – Impute* | 3.2 GB | 3 | Chr 1 | 0:44:52 | 0:44:52 |
| Step 3b - Convert | 3.7 GB | 3 | HGDP Dataset | 1:29:32 | 1:29:32 |
| Step 4 - Analyze | 4.8 GB | 3 | HGDP Dataset | 0:11:25 | 0:11:25 |
| Step 4 - Visualize | 17.7 GB | 1 | HGDP Dataset | 0:12:45 | 0:12:45 |
| Pop Strat Add-in | 17.2 GB | 1 | HGDP Target – 1K Genomes Reference | 0:18:42 | 0:18:42 |
| Total Estimation | 17.7 GB | - | - | - | 3:00:41 |
| **Eagle2-Minimac4 Workflow | | | | | |

| Step | Max Memory Required | CPU's Used | Benchmarking Sample | Time per Job on HPS | Time per Step on HPS |
| --- | --- | --- | --- | --- | --- |
| Step 0 - Clean-up | 0.9 GB | 1 | HGDP Dataset | 0:18:15 | 0:18:15 |
| Step 1 - Pre-QC | 0.1 GB | 1 | HGDP Dataset | 0:01:07 | 0:01:07 |
| Step 2 – Phase* | 1.5 GB | 3 | Chr 1 | 1:27:53 | 1:27:53 |
| Step 3a – Impute* | 1.7 GB | 1 | Chr 1 Segment 1 | 0:05:38 | 0:18:31 |
| Step 3b - Convert | 2.2 GB | 3 | HGDP Dataset | 5:14:40 | 5:14:40 |
| Step 4 - Analyze | 4.8 GB | 3 | HGDP Dataset | 0:11:25 | 0:11:25 |
| Step 4 - Visualize | 17.7 GB | 1 | HGDP Dataset | 0:12:45 | 0:12:45 |
| Pop Strat Add-in | 17.2 GB | 1 | HGDP Target –  1K Genomes Reference | 0:18:42 | 0:18:42 |
| Total Estimation | 17.7 GB | - | - | - | 8:03:18 |
| **SHAPEIT2-IMPUTE4 Workflow | | | | | |


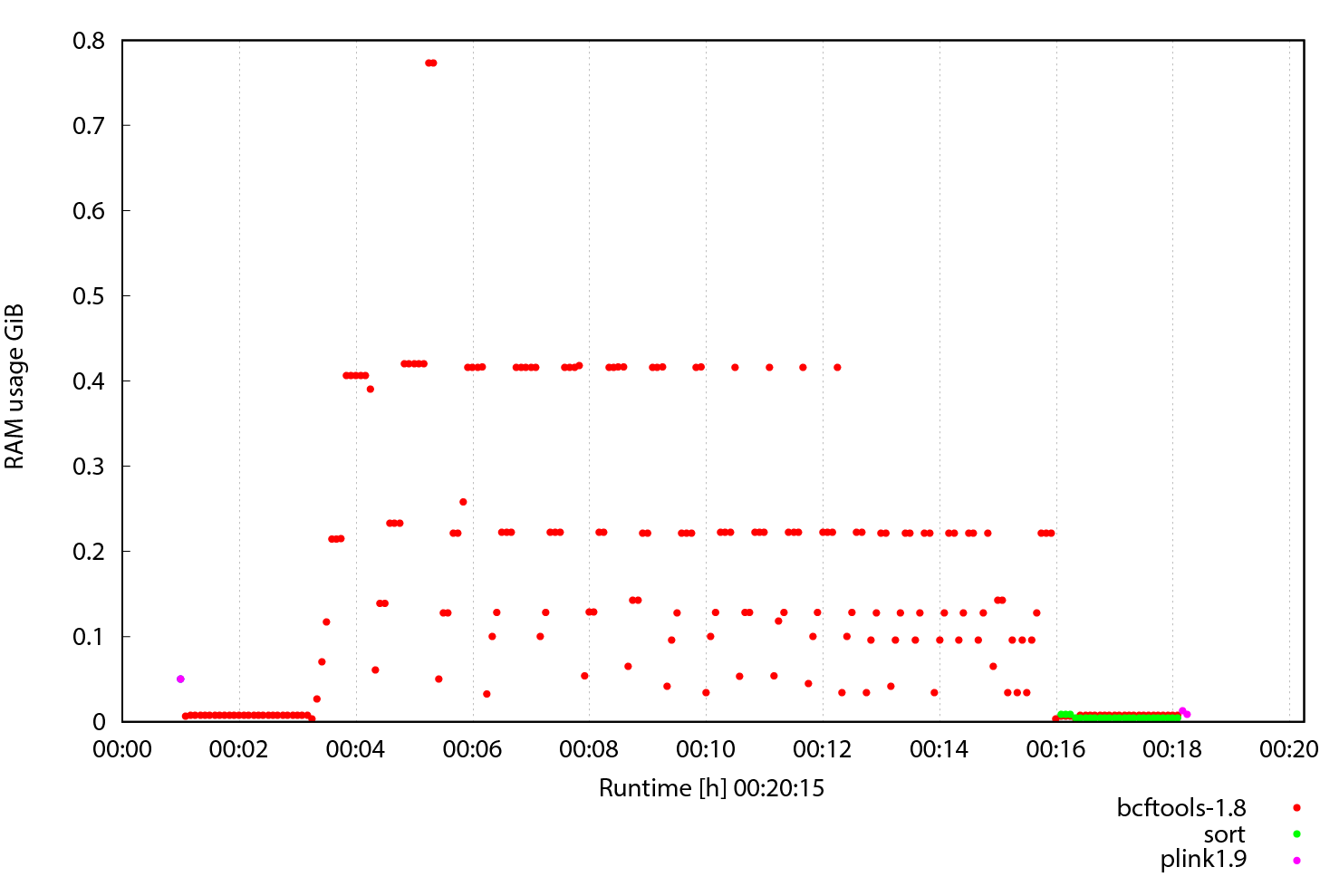

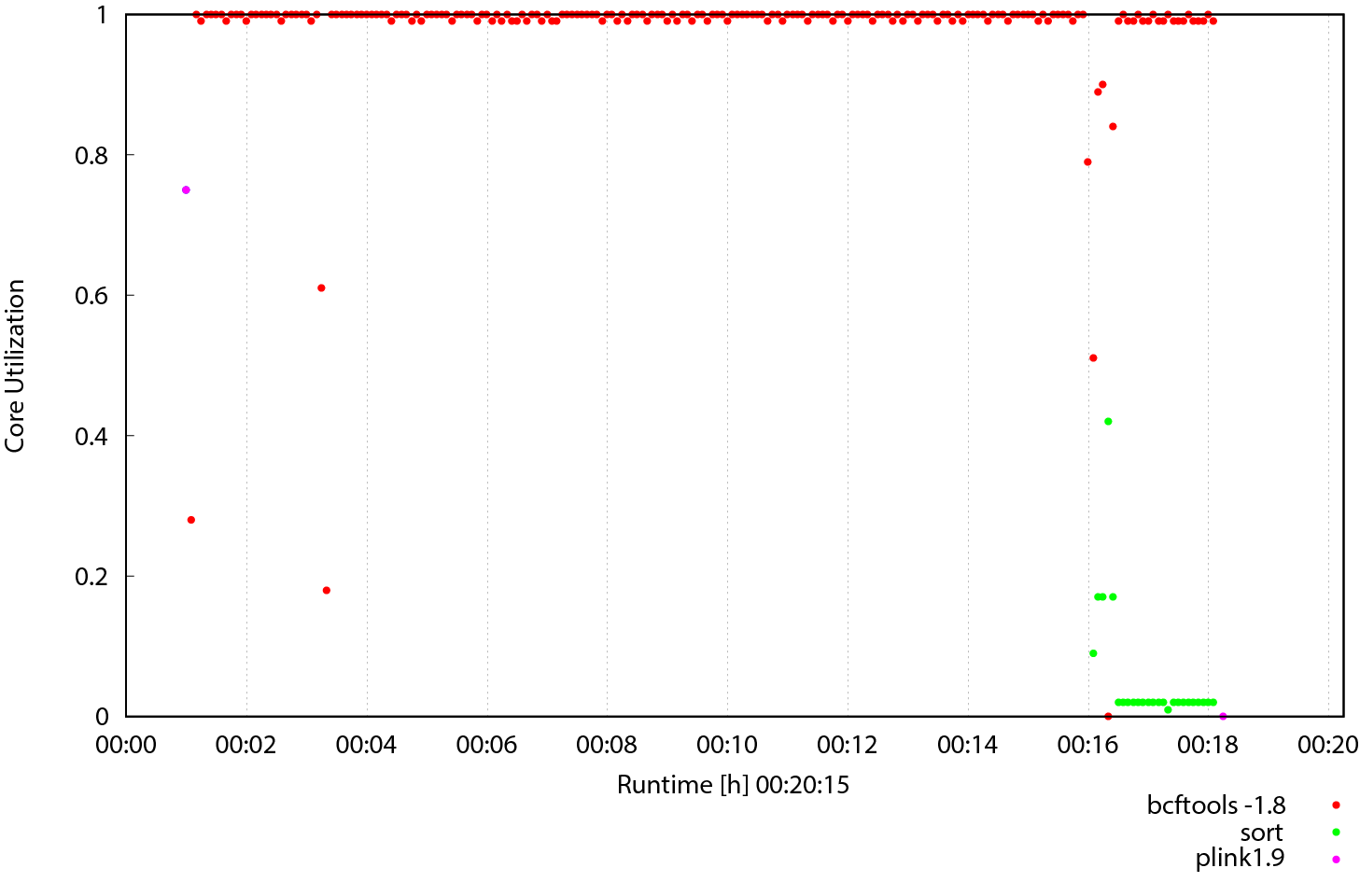


**Figure S1. CPU and Ram Utilization for Step 0 – Data Cleanup.** Odyssey Step 0, which fixes strand orientation of the user’s dataset to a reference genome, was performed on the entire HGDP dataset containing 940 admixed individuals and approximately 542K markers. Collectl was used to monitor the CPU (left) and RAM (right) usage for each process (i.e. program) in the step as well as the total time for completion. A buffer of 120 seconds was added to the end of the step for technical reasons. 1 hyperthreaded CPU core and 32 GB RAM were allotted for Step 0. Using the entire specified dataset, the data cleanup step would require approximately 18 minutes and 1 GB RAM.


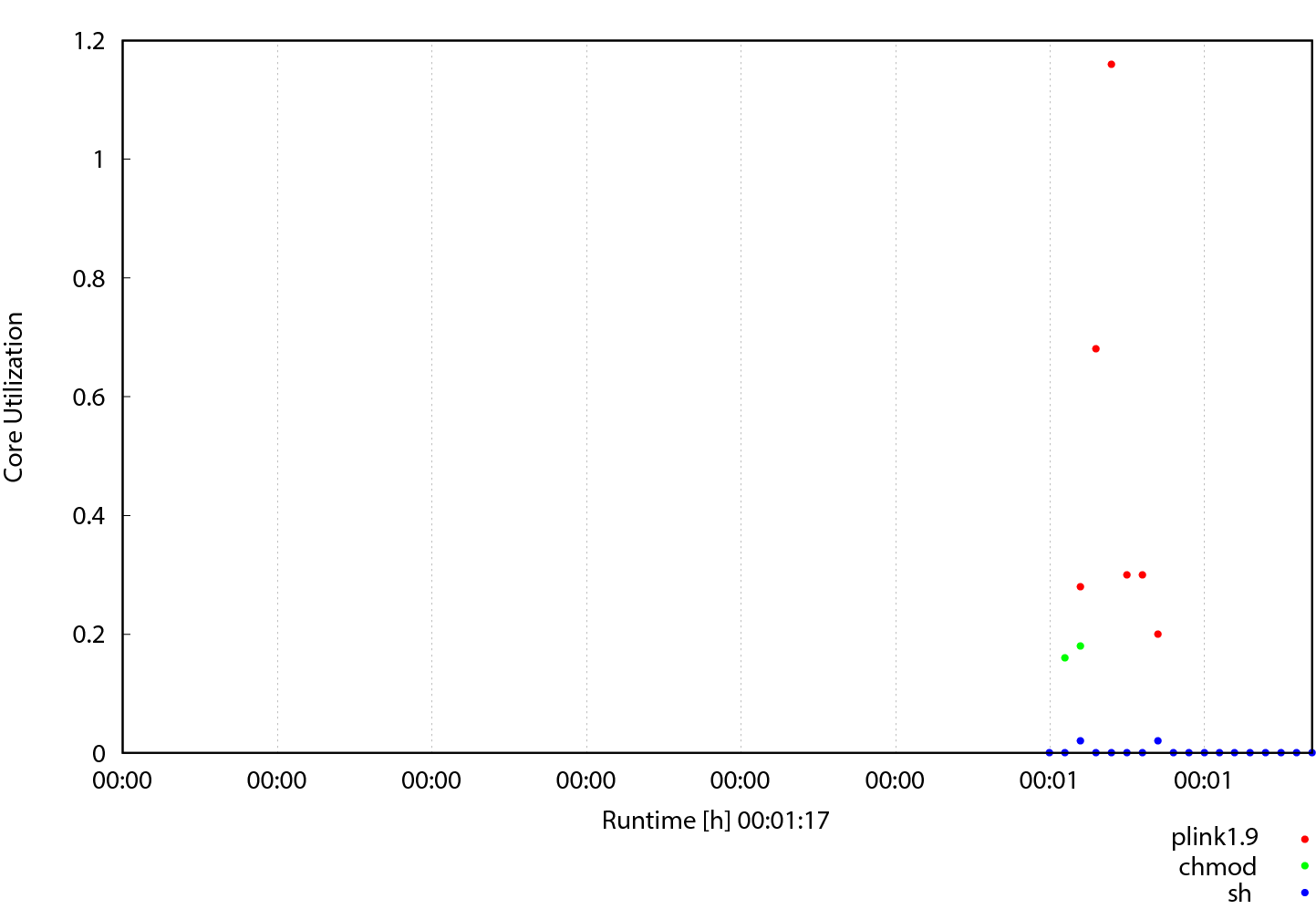

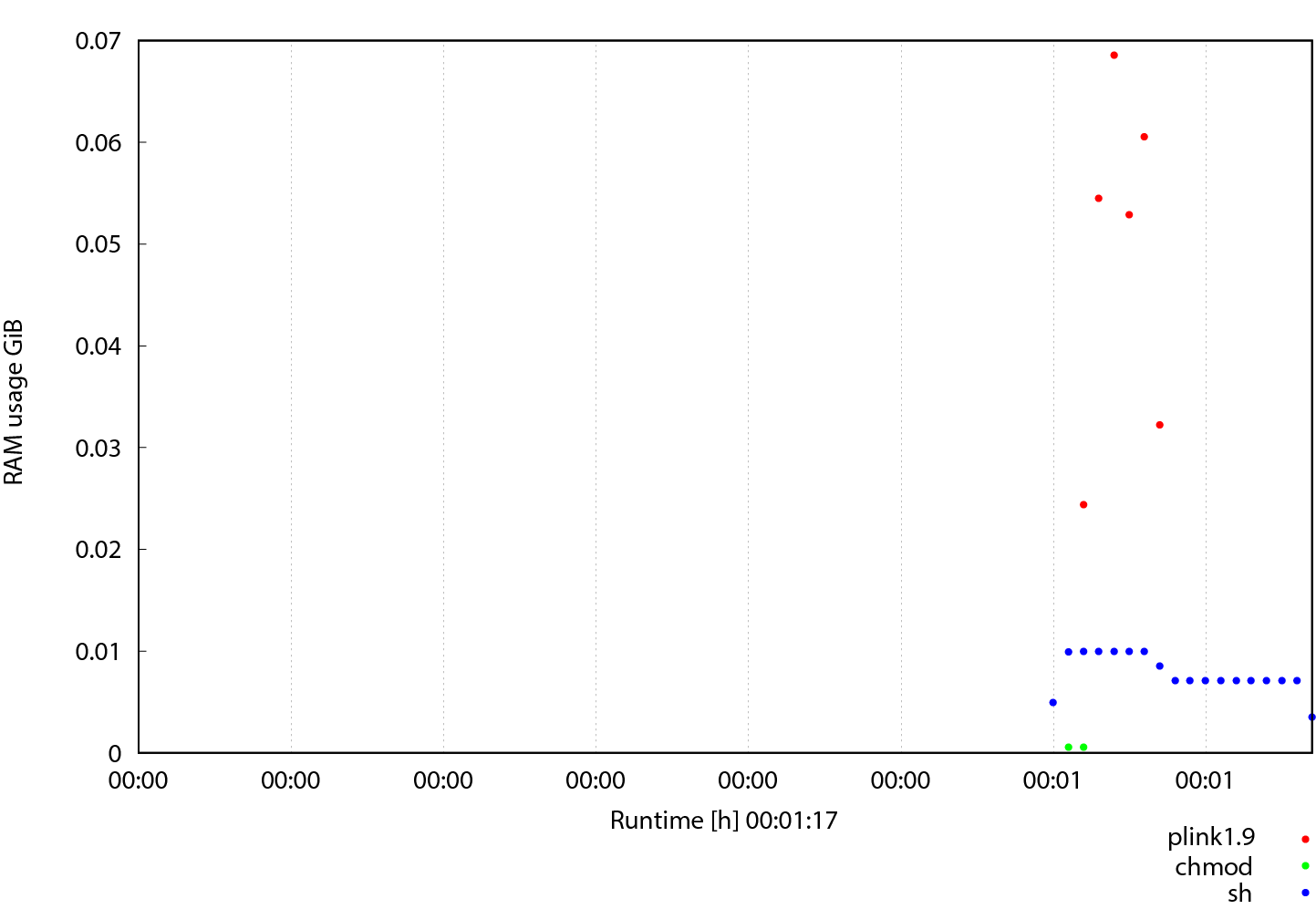


**Figure S2: CPU and Ram Utilization for Step 1 – Data Quality Control.** Odyssey Step 1, which performs quality control metrics of missingness, minor allele frequency, and Hardy-Weinberg equilibrium filters as well as divide the dataset into individual chromosomes, was performed on the entire HGDP dataset containing 940 admixed individuals and approximately 542K markers. Collectl was used to monitor the CPU (left) and RAM (right) usage for each process (i.e. program) in the step as well as the total time for completion. A buffer of 10 seconds was added to the end of the step for technical reasons. 1 hyperthreaded CPU core and 32 GB RAM were allotted for Step 1. Using the entire specified dataset, the data quality control step would require approximately 1 minute and <1 GB RAM.


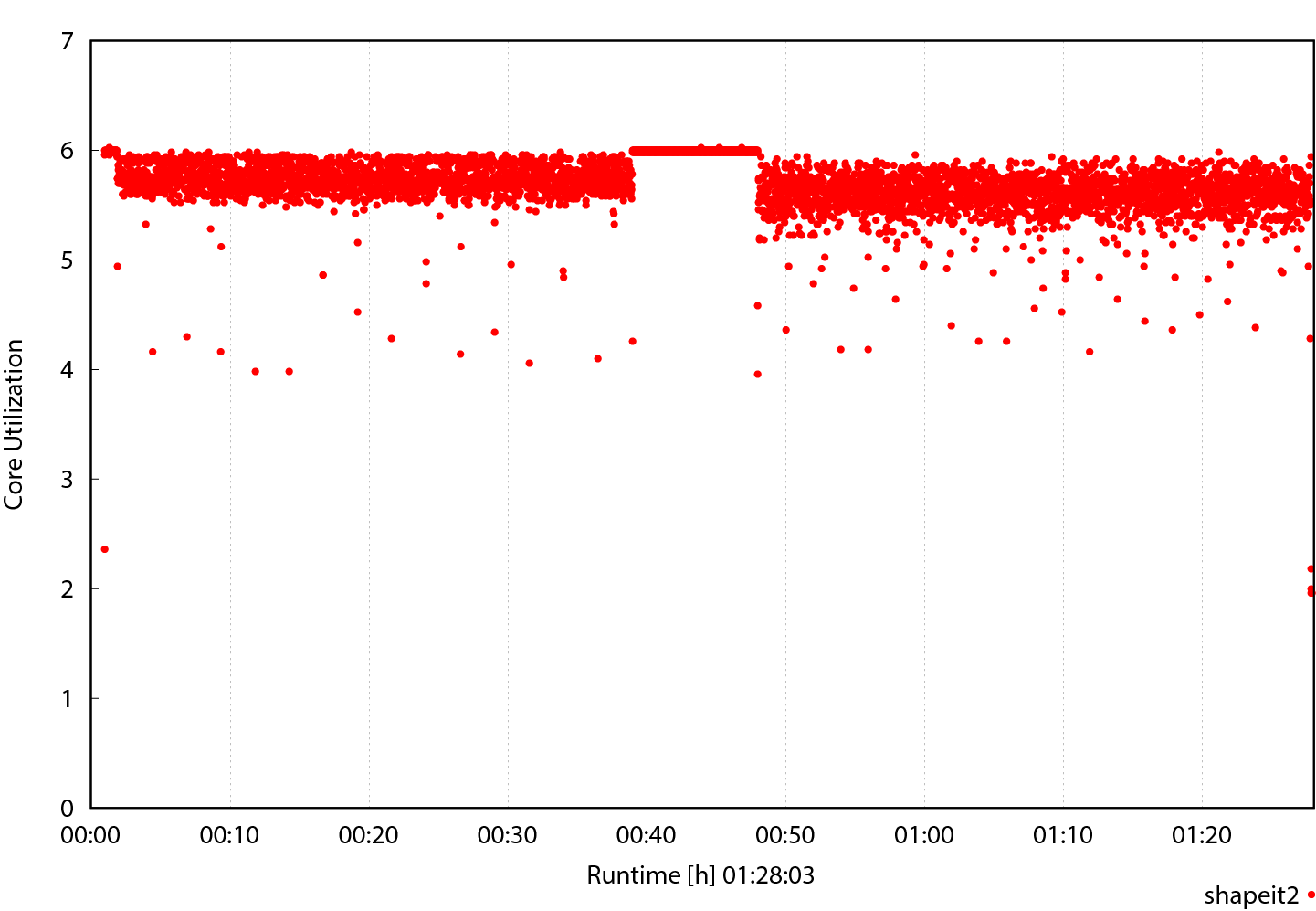

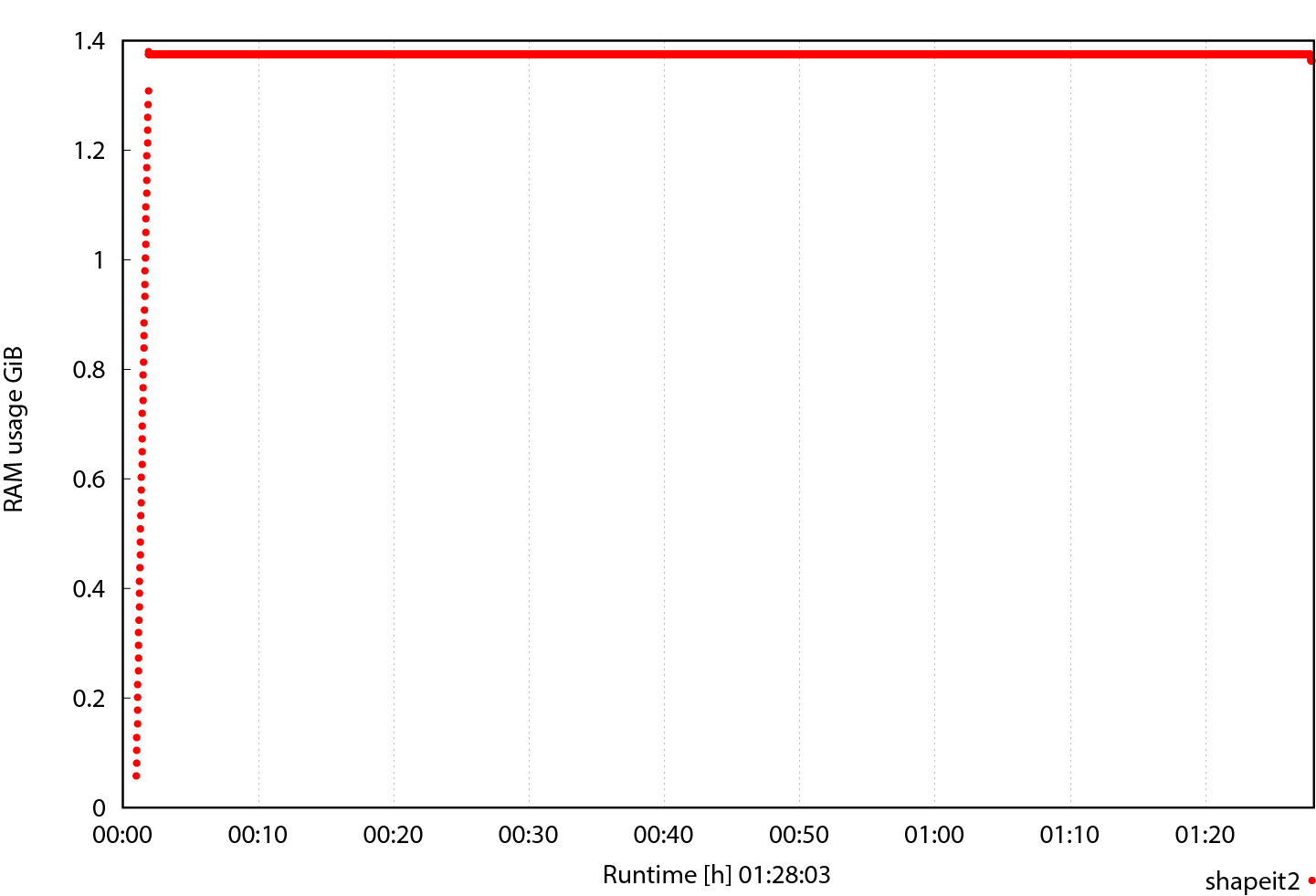


**Figure S3: CPU and Ram Utilization for Step 2 – Phasing [SHAPEIT-IMPUTE Workflow].** Odyssey Step 2, which performs phasing via SHAPEIT2, was performed on chromosome 1 of the HGDP dataset containing 940 admixed individuals and approximately 542K markers (30893 markers were phased on Chromosome 1). Collectl was used to monitor the CPU (left) and RAM (right) usage for each process (i.e. program) in the step as well as the total time for completion. A buffer of 10 seconds was added to the end of the step for technical reasons. 3 hyperthreaded CPU cores were allotted to SHAPEIT2 (which was programmed to run on 6 threads) as well as 32 GB RAM. Phasing 1 chromosome from the specified dataset would require approximately 1.5 hours and 1.5 GB RAM.


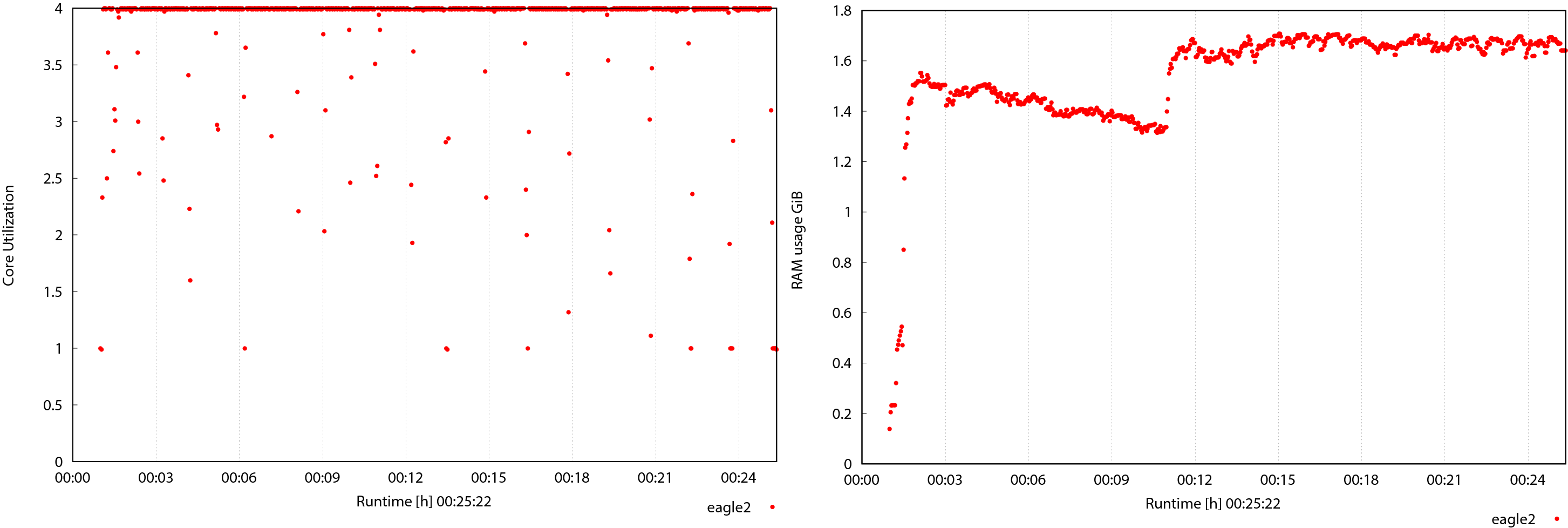
**Figure S4: CPU and Ram Utilization for Step 2 – Phasing [Eagle-Minimac Workflow].** Odyssey Step 2, which performs phasing via Eagle2, was performed on chromosome 1 of the HGDP dataset containing 940 admixed individuals and approximately 542K markers (36543 markers were phased on Chromosome 1). Collectl was used to monitor the CPU (left) and RAM (right) usage for each process (i.e. program) in the step as well as the total time for completion. 3 hyperthreaded CPU cores were allotted to Eagle2 (which was programmed to run on 6 threads) as well as 32 GB RAM. Phasing 1 chromosome from the specified dataset would require approximately 24 minutes and 1.9 GB RAM.


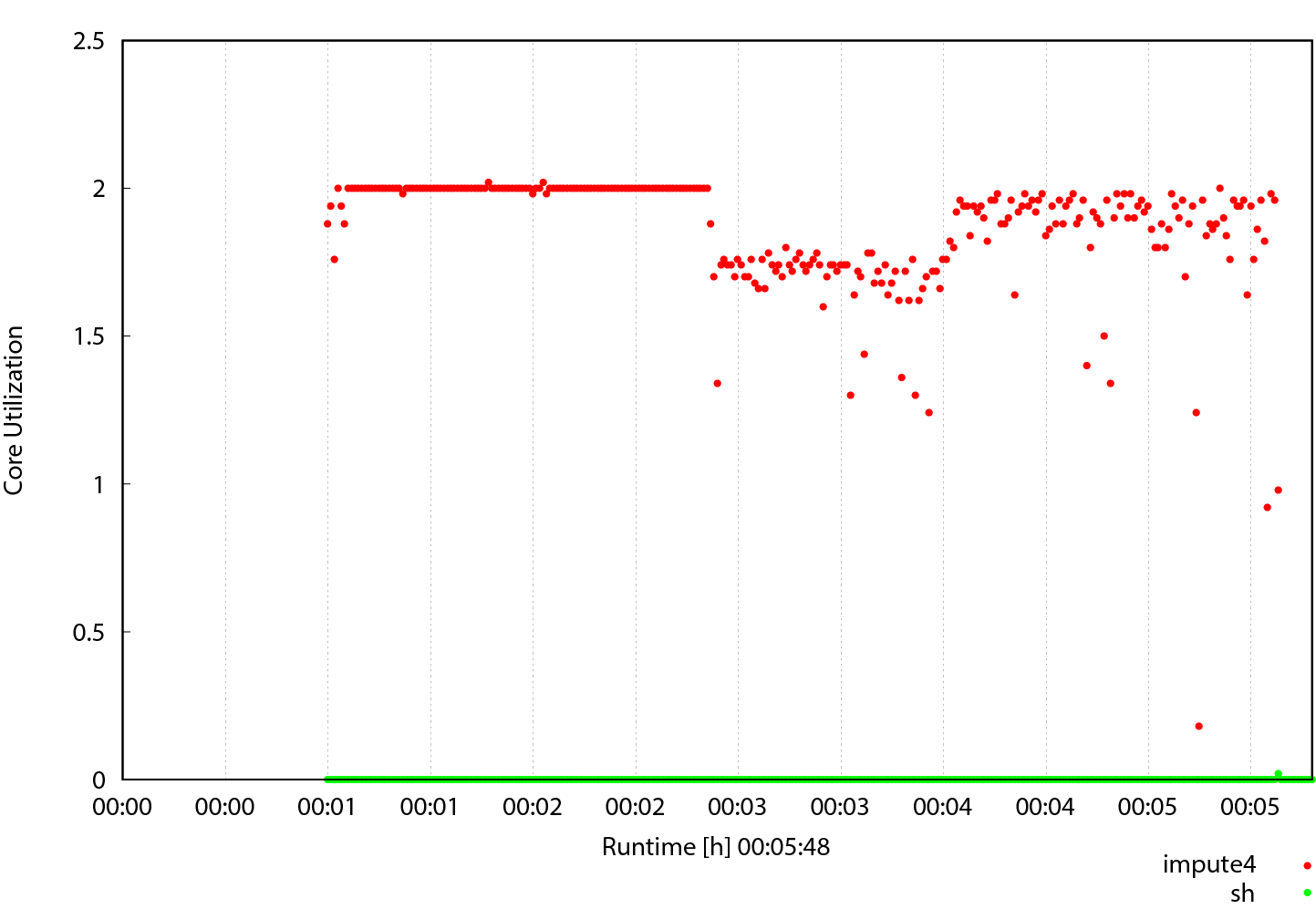

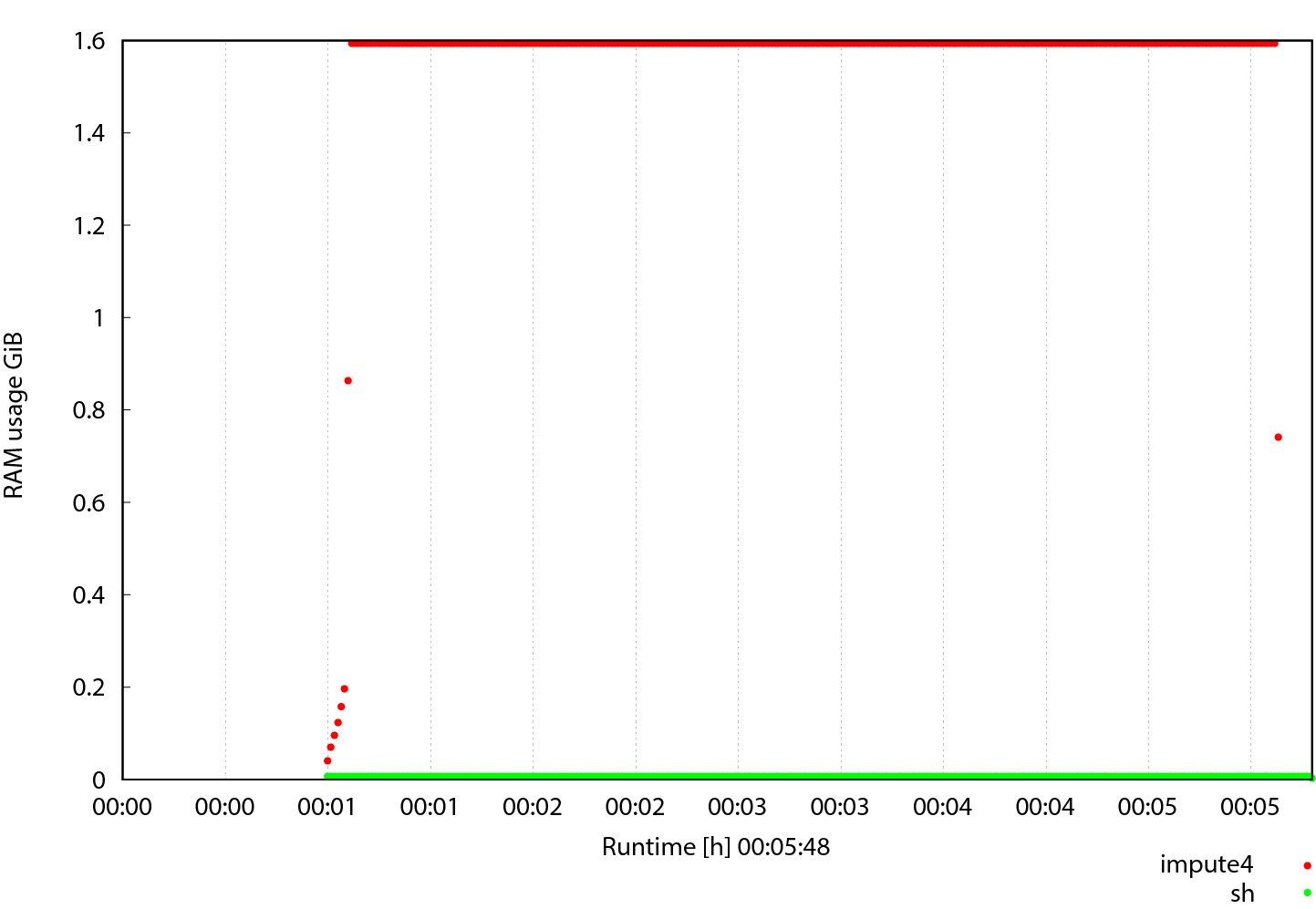


**Figure S5: CPU and Ram Utilization for Step 3a – Imputation [SHAPEIT-IMPUTE Workflow].** Odyssey Step 3a, which performs imputation via IMPUTE4, was performed on the first 5 megabase segment of chromosome 1 of the HGDP dataset containing 940 admixed individuals and approximately 542K markers (4316 phased markers were used for imputation on the first 5MB segment on Chromosome 1 resulting in 161268 total imputed and genotyped markers). Collectl was used to monitor the CPU (left) and RAM (right) usage for each process (i.e. program) in the step as well as the total time for completion. A buffer of 10 seconds was added to the end of the step for technical reasons. 1 hyperthreaded CPU core and 32 GB RAM was allotted to IMPUTE4 for Step 3a. Imputing the first 5 megabase segment of 1 chromosome from the specified dataset would require approximately 6 minutes and 1.7 GB RAM.


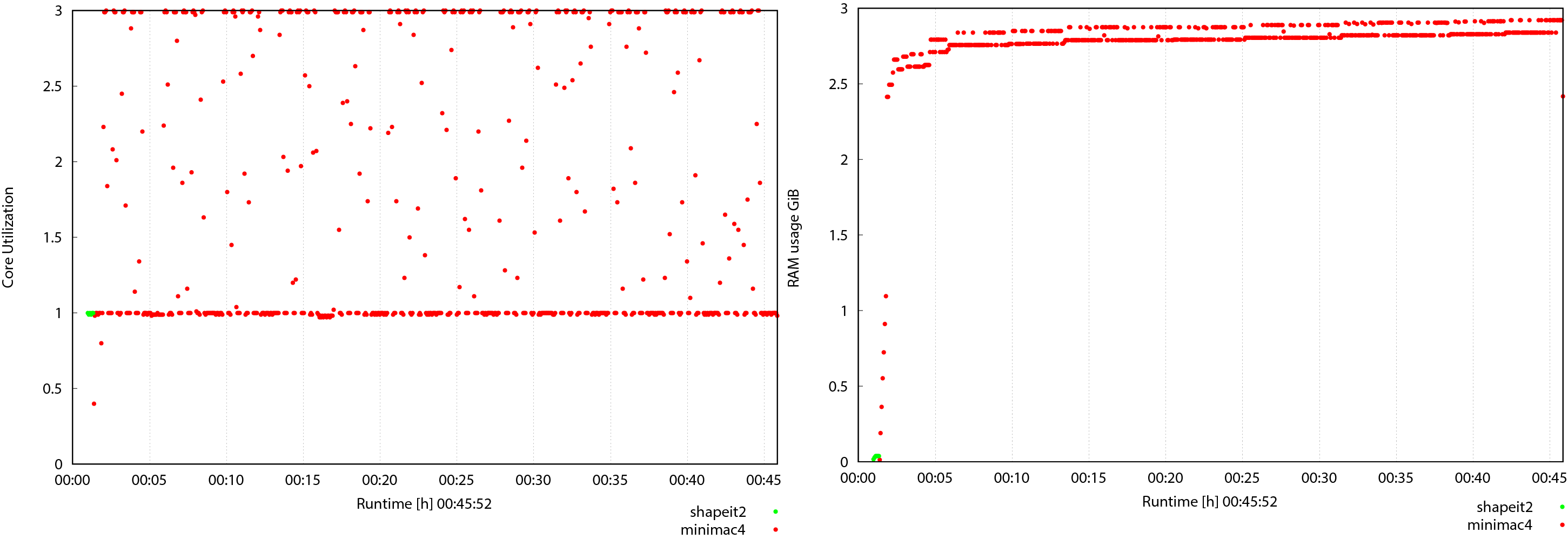
**Figure S6: CPU and Ram Utilization for Step 3a – Imputation [Eagle-Minimac Workflow].** Odyssey Step 3a, which performs imputation via Minimac4, was performed on chromosome 1 of the HGDP dataset containing 940 admixed individuals and approximately 542K markers (36543 Eagle2 phased markers were used for imputation on Chromosome 1 resulting in 3745840 total imputed and genotyped markers). Collectl was used to monitor the CPU (left) and RAM (right) usage for each process (i.e. program) in the step as well as the total time for completion. 3 hyperthreaded CPU cores and 32 GB RAM was allotted to Minimac4 (which was programmed to run on 6 threads) for Step 3a. Imputing Chromosome 1 from the specified dataset would require approximately 44 minutes and 3.2 GB RAM.


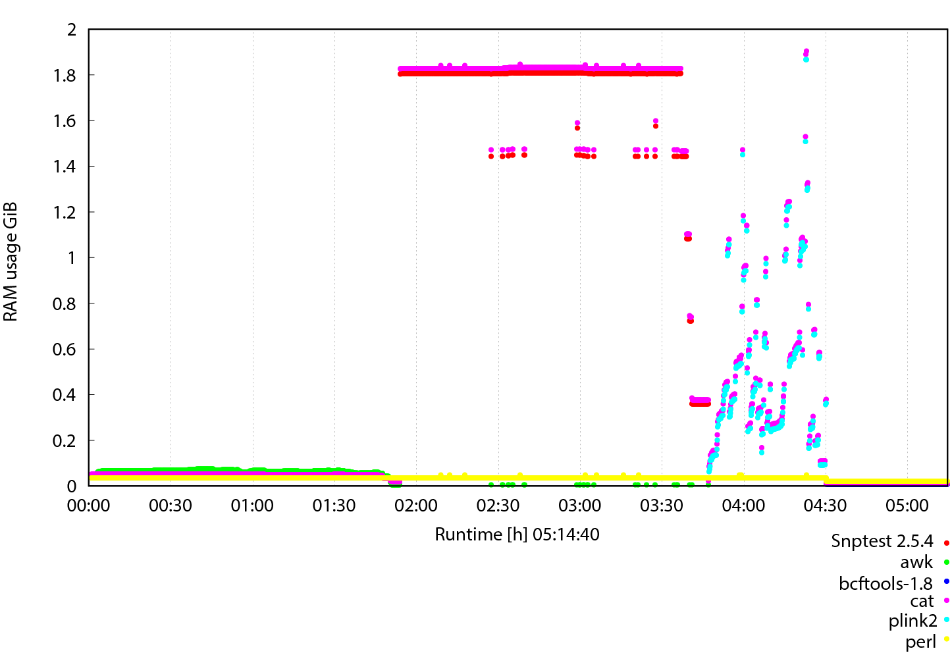

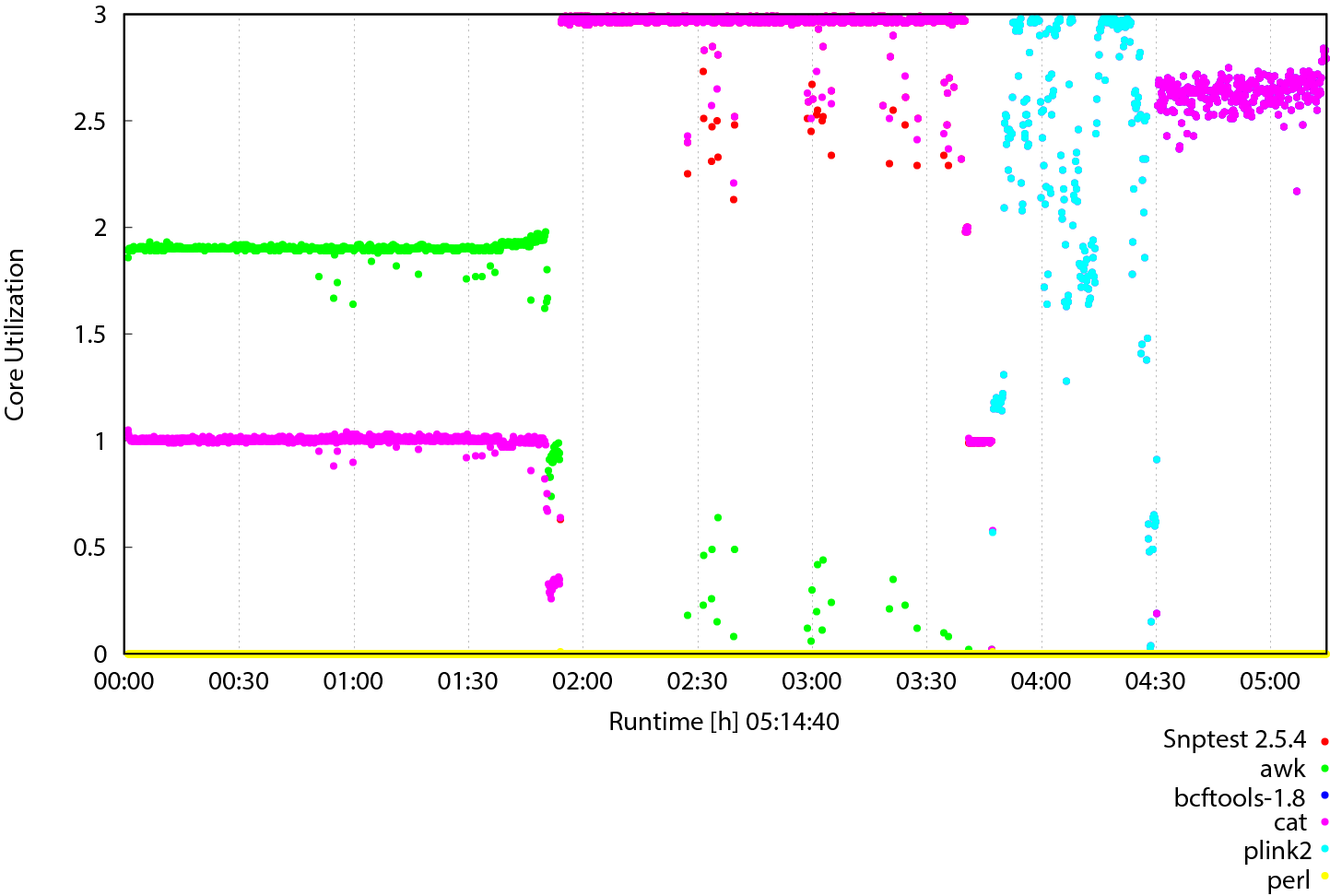


**Figure S7: CPU and Ram Utilization for Step 3b – Concatenation, Post-Imputation Quality Control, and Conversion [SHAPEIT-IMPUTE Workflow].** Odyssey Step 3b calculates post-imputation quality control metrics via SNPTEST, converts the segmented chromosomal .gen files to VCF files while filtering based on a specified QC metric (i.e. the INFO metric since IMPUTE4 is being used), and concatenates the chromosomal VCF files file for analysis. This step was performed on the entire HGDP dataset containing 940 admixed individuals and approximately 39 million imputed and genotyped markers after post-imputation quality-control measures. Collectl was used to monitor the CPU (left) and RAM (right) usage for each process (i.e. program) in the step as well as the total time for completion. 3 hyperthreaded CPU cores and 32 GB RAM were allotted for Step 3b. Using the entire specified dataset, Step 3b would require approximately 5.2 hours and 2 GB RAM. Note that BCFTools and cat are overlapped on the core utilization figure between time points 4:30 and 5:14.


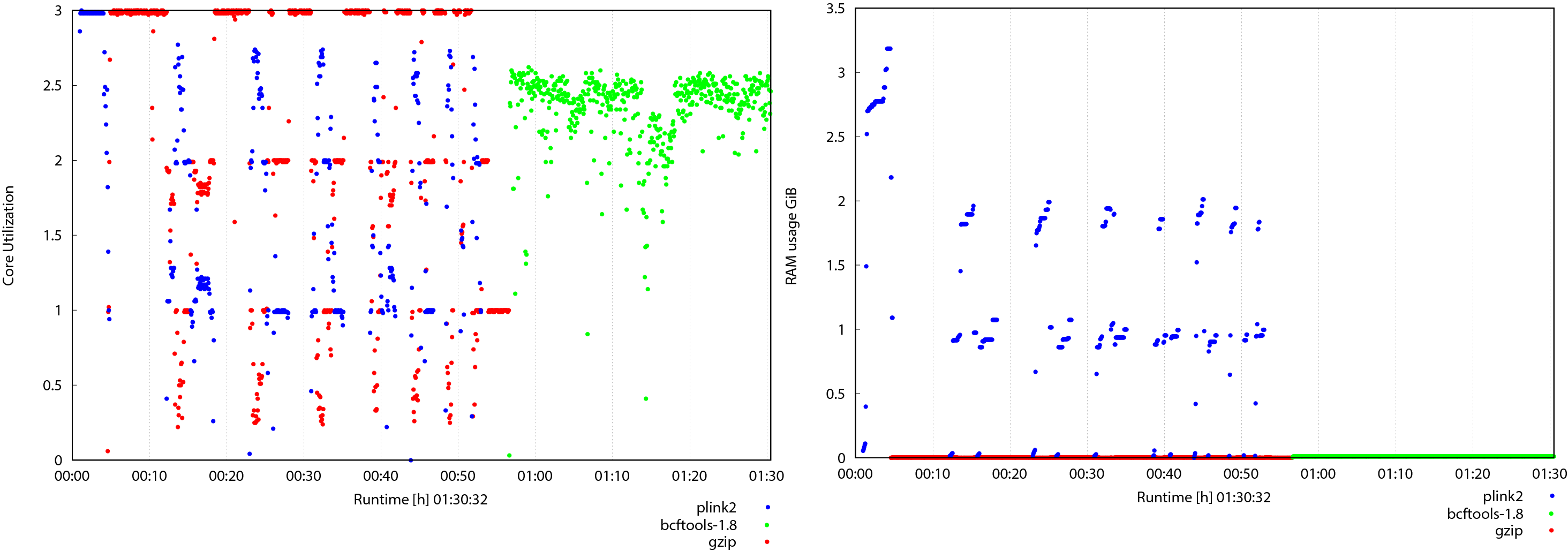
**Figure S8: CPU and Ram Utilization for Step 3b – Concatenation, Post-Imputation Quality Control, and Conversion [Eagle-Minimac Workflow].** Odyssey Step 3b filters the dosage.vcf.gz based on a specified QC metric (i.e. the R^2^ metric since Minimac4 is being used), converts, and then merges all chromosomal dosage .vcf.gz files into a single dosage vcf.gz file for analysis. This step was performed on the entire HGDP dataset containing 940 admixed individuals and approximately 25.4 million imputed and genotyped markers after post-imputation quality-control measures. Collectl was used to monitor the CPU (left) and RAM (right) usage for each process (i.e. program) in the step as well as the total time for completion. 3 hyperthreaded CPU cores and 32 GB RAM were allotted for Step 3b. Using the entire specified dataset, Step 3b would require approximately 1.5 hours and 3.7 GB RAM.


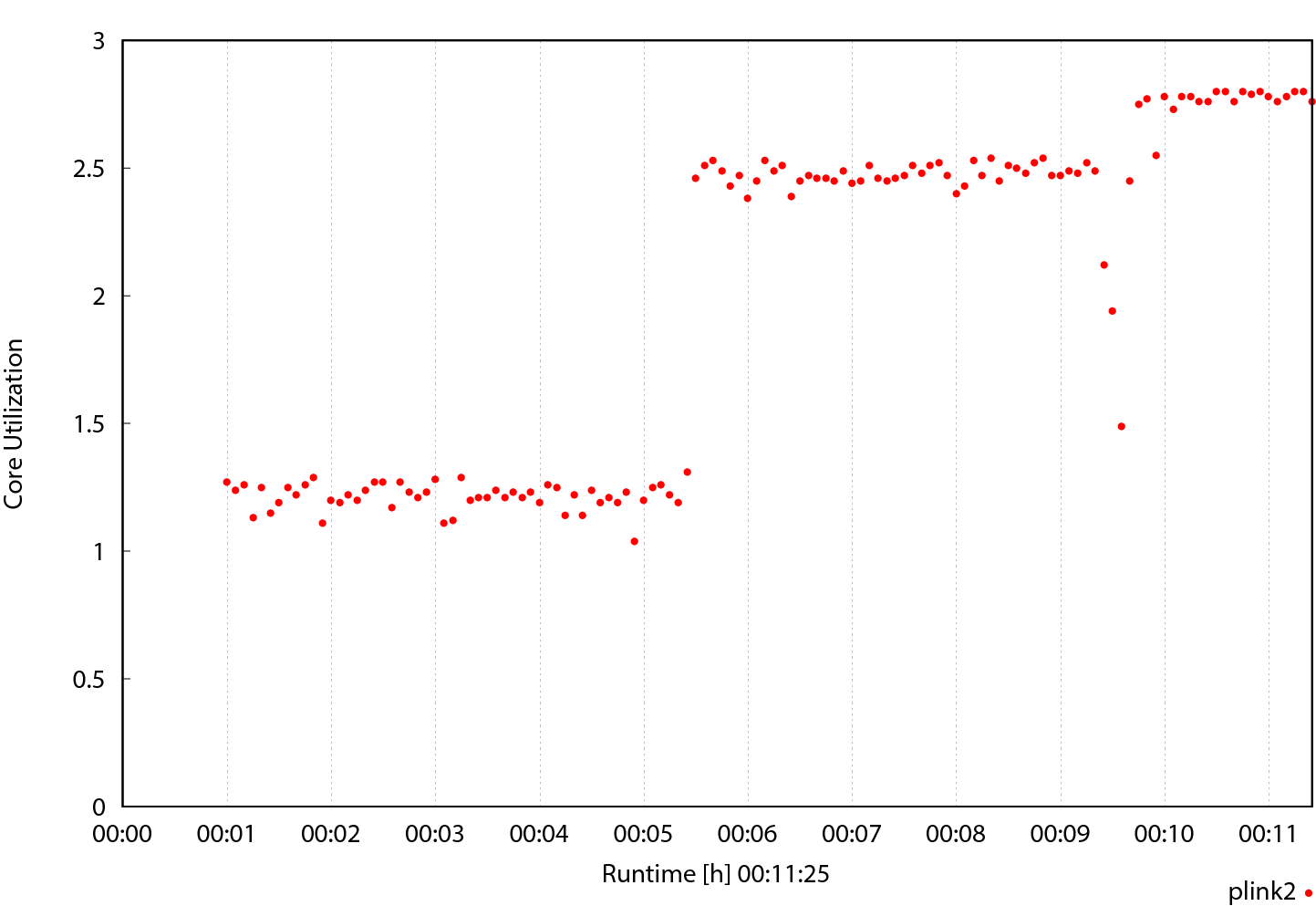

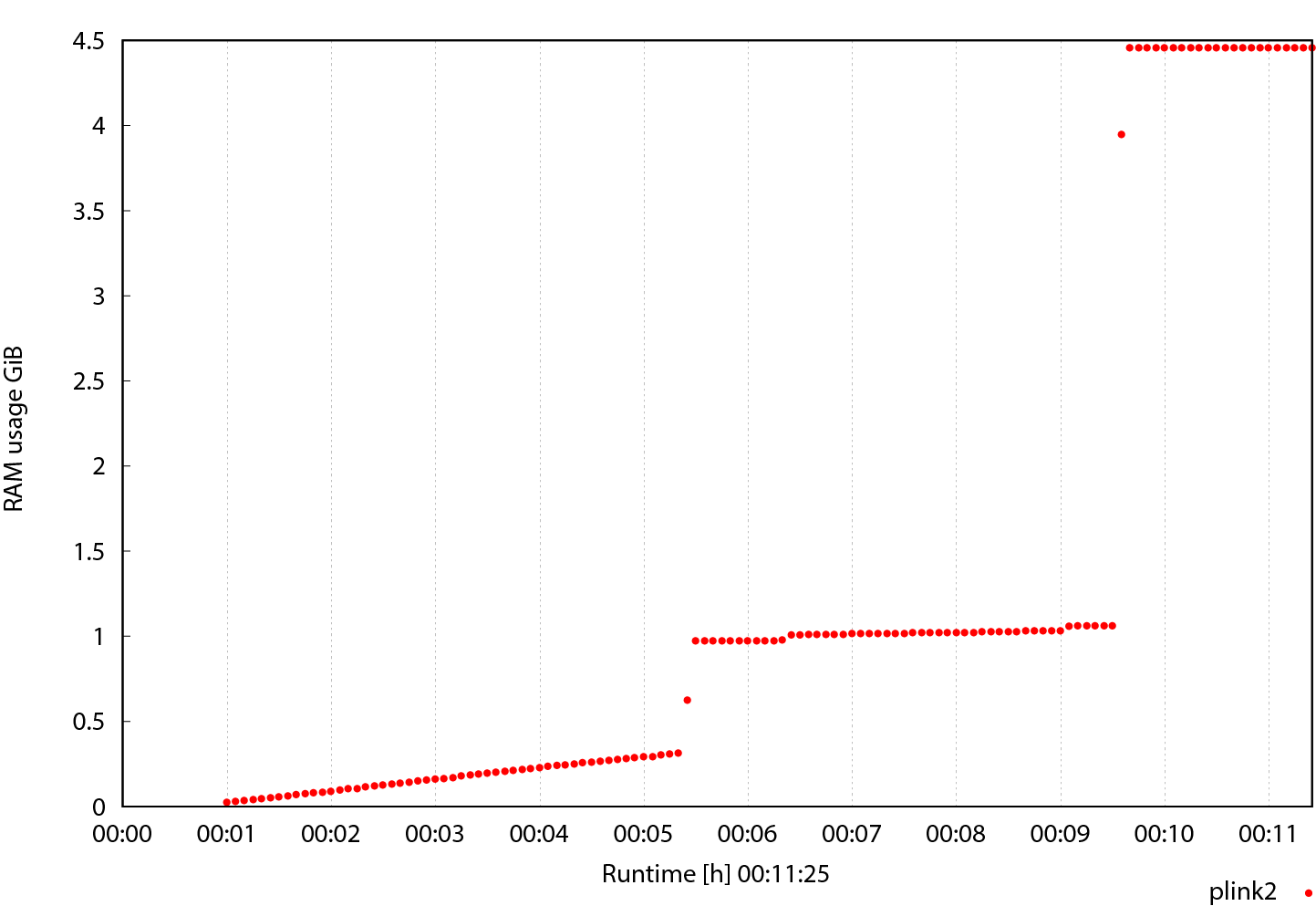


**Figure S9: CPU and Ram Utilization for Step 4 – Analysis.** Odyssey Step 4 performs a simple Genome-Wide-Association test by using PLINK2 to create a general linear regression model on the 39 million imputed and genotyped variants (generated from the SHAPEIT-IMPUTE workflow) in 940 admixed individuals using sex as a covariate and using randomly calculated phenotype data that contains values from zero to one. 3 hyperthreaded CPU cores and 32 GB RAM were allotted for Step 4. Collectl was used to monitor the CPU (left) and RAM (right) usage for each process (i.e. program) in the step as well as the total time for completion. Using the entire specified dataset, analysis would require approximately 11.5 minutes and 4.5 GB RAM.


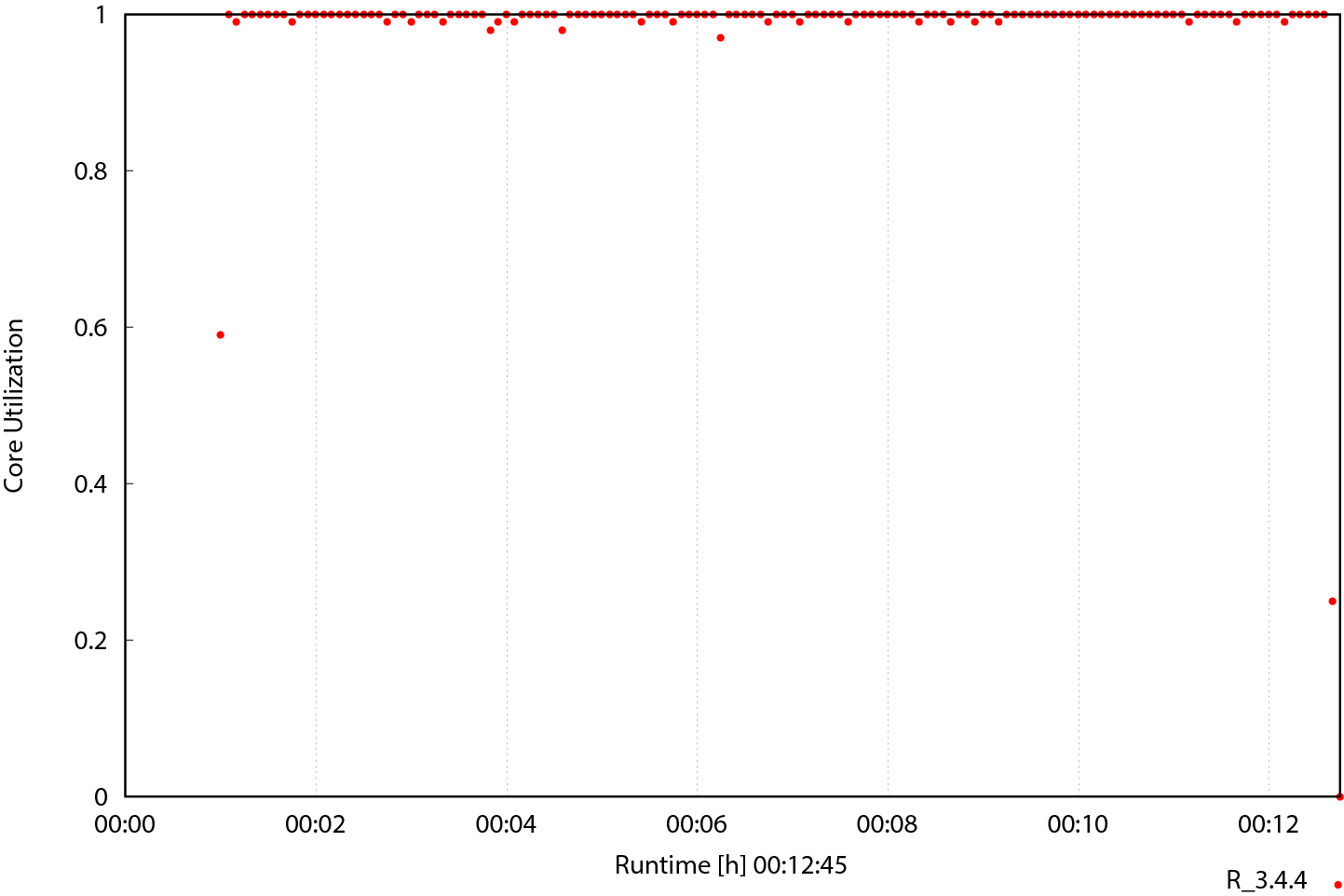

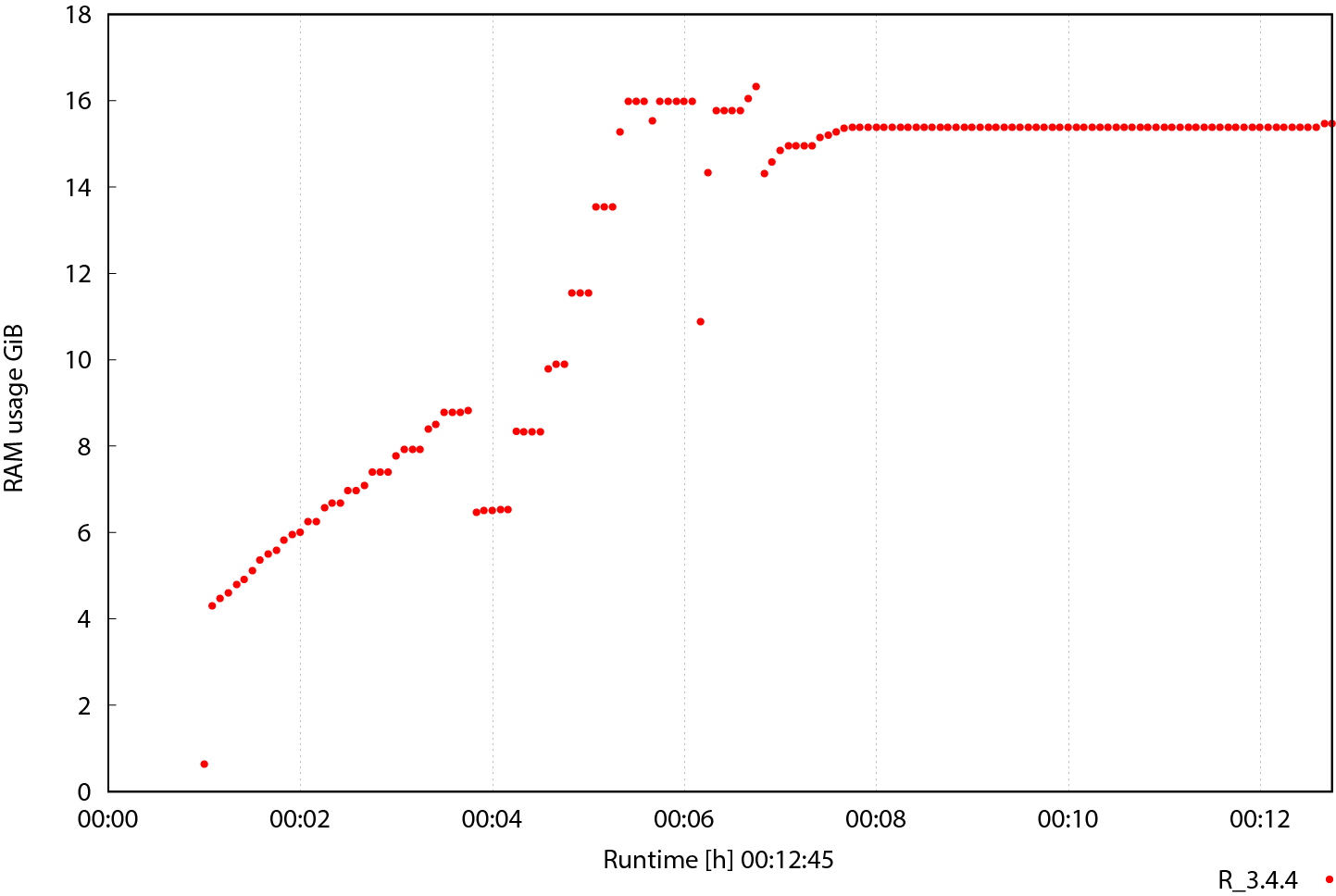


**Figure S10: CPU and Ram Utilization for Step 4 – Visualization.** Odyssey Step 4 uses R-3.4.4 to read and clean the results file output from the analysis portion of Step 4. R performs a Bonferroni and Benjamini-Hochberg procedure to account for multiple comparisons, sorts the data, and outputs 1) a sorted table of the top 10000 variants with the smallest unadjusted p-values, 2) a qqPlot, and 3) an interactive Manhattan plot. The raw data file is then gunzipped. 1 hyperthreaded CPU and 32 GB RAM were allotted for Step 4. Collectl was used to monitor the CPU (left) and RAM (right) usage for each process (i.e. program) in the step as well as the total time for completion. Step 3b would require approximately 13 minutes and 18 GB RAM.


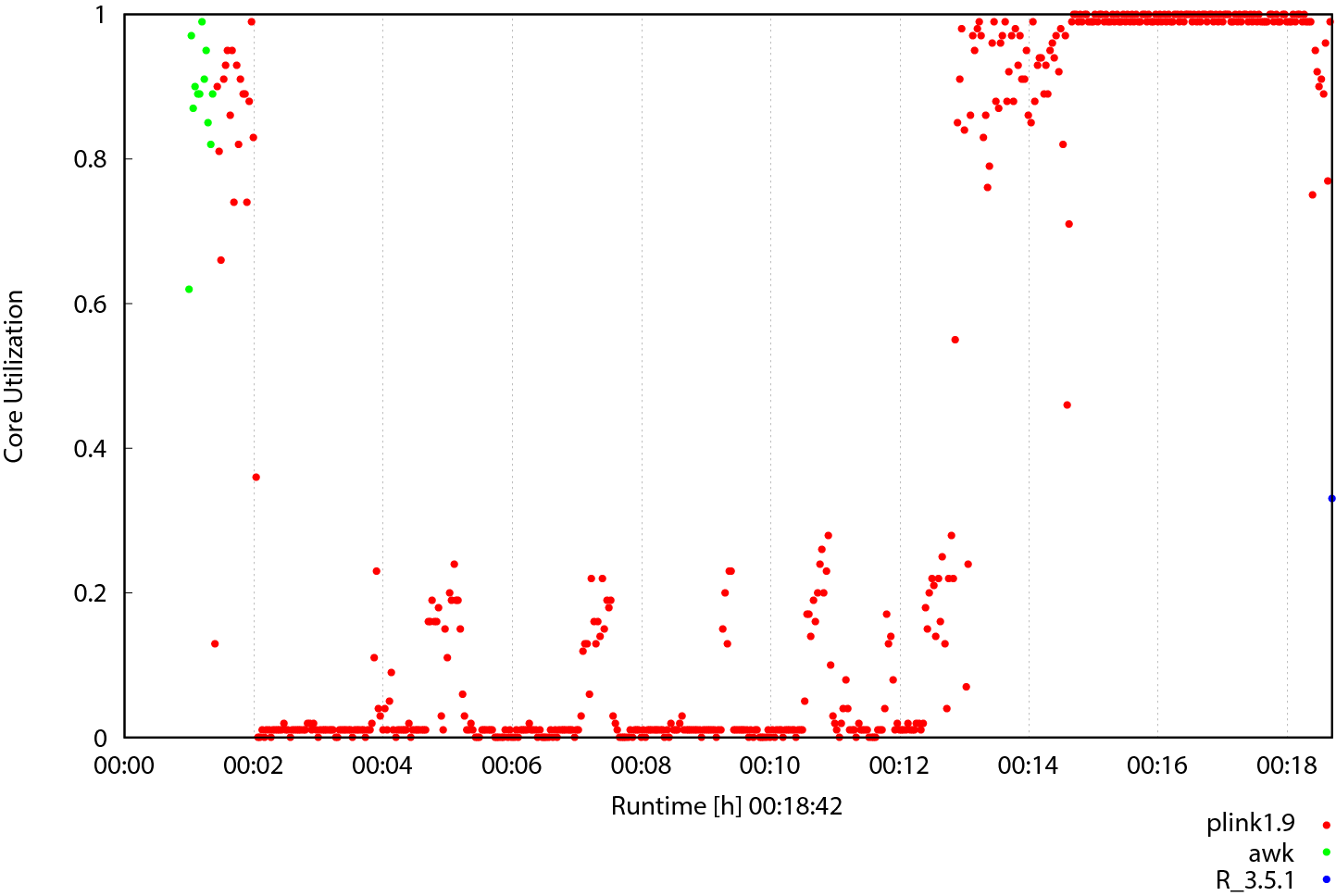

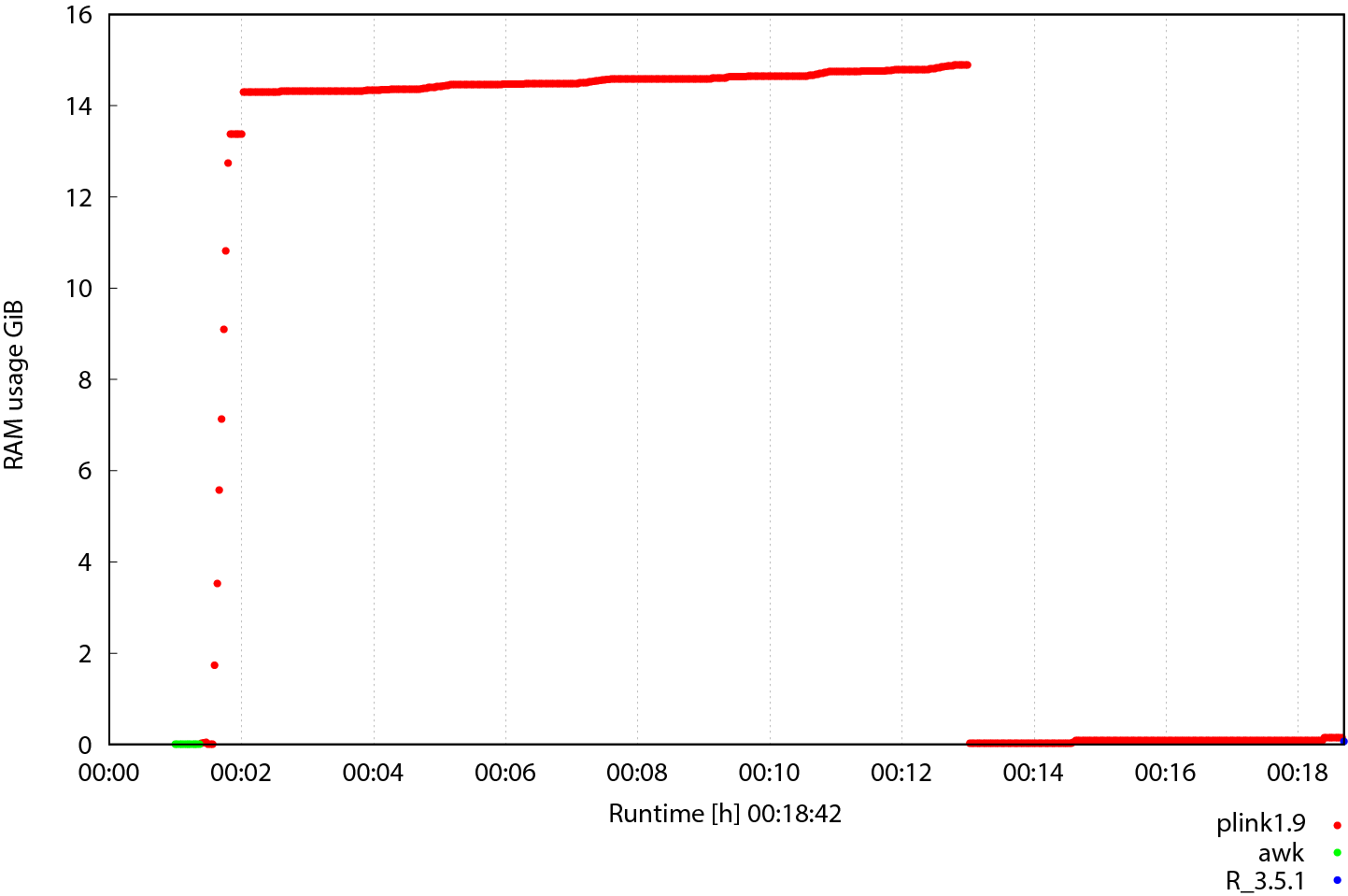


**Figure S11: CPU and Ram Utilization for Population Stratification Module.** The optional Population Stratification Module uses an admixed reference set of known ancestries to predict the ancestries of a target dataset via a Principal Component analysis (PCA), uses a user-defined subset of reference data corresponding to an ancestry of interest to calculate an “ancestral centroid”, which is then used to remove individuals falling outside of the desired ancestral group. R-3.5.1 is used to visualize the PCA including individuals kept for GWAS, removed due to being ancestral outliers, and those that were used as a reference. The processed PCA results in addition to a list of individuals who should be dropped from the GWAS is also output by R. In this benchmark the 1000 Genomes Phase 3 dataset was used as a reference, the 940 admixed individual HGDP dataset was used as the target dataset, the ancestral group of interest that was selected was European, principal components that contributed 1% or more were selected for centroid creation, and individuals that fell outside of 3 standard deviations of the centroid’s dimensions were considered ancestral outliers and removed. 1 hyperthreaded CPU and 32 GB RAM were allotted to run the Population Stratification add-in. Collectl was used to monitor the CPU (left) and RAM (right) usage for each process (i.e. program) as well as the total time for completion. The add-in would require approximately 19 minutes and 18 GB RAM.
